# SILAC kinase screen identifies potential MASTL substrates

**DOI:** 10.1038/s41598-022-14933-0

**Published:** 2022-06-22

**Authors:** Kamila A. Marzec, Samuel Rogers, Rachael McCloy, Benjamin L. Parker, David E. James, D. Neil Watkins, Andrew Burgess

**Affiliations:** 1 ANZAC Research Institute, Concord Hospital, Concord, NSW 2139 Australia; 2 Children’s Medical Research Institute, The University of Sydney, Westmead, Australia; 3 The Kinghorn Cancer Centre, Garvan Institute of Medical Research, Sydney, NSW 2010 Australia; 4 Department of Anatomy and Physiology, University of Melbourne, Melbourne, VIC 3010 Australia; 5 Charles Perkins Centre, School of Life and Environmental Sciences, The University of Sydney, Sydney, NSW 2006 Australia; 6 School of Medical Sciences, The University of Sydney, Sydney, NSW 2006 Australia; 7 Department of Internal Medicine, Rady Faculty of Health Sciences, University of Manitoba, Winnipeg, MB R3E 0W2 Canada; 8 CancerCare Manitoba Research Institute, Winnipeg, MB R3E-0V9 Canada; 9 The University of Sydney Concord Clinical School, Faculty of Medicine and Health, Sydney, NSW 2139 Australia

**Keywords:** Proteomics, Cell division, Mass spectrometry, Proteomic analysis, Cell signalling

## Abstract

Microtubule-associated serine/threonine kinase-like (MASTL) has emerged as a critical regulator of mitosis and as a potential oncogene in a variety of cancer types. To date, Arpp-19/ENSA are the only known substrates of MASTL. However, with the roles of MASTL expanding and increased interest in development of MASTL inhibitors, it has become critical to determine if there are additional substrates and what the optimal consensus motif for MASTL is. Here we utilized a whole cell lysate in vitro kinase screen combined with stable isotope labelling of amino acids in cell culture (SILAC) to identify potential substrates and the residue preference of MASTL. Using the related AGC kinase family members AKT1/2, the kinase screen identified several known and new substrates highly enriched for the validated consensus motif of AKT. Applying this method to MASTL identified 59 phospho-sites on 67 proteins that increased in the presence of active MASTL. Subsequent in vitro kinase assays suggested that MASTL may phosphorylate hnRNPM, YB1 and TUBA1C under certain in vitro conditions. Taken together, these data suggest that MASTL may phosphorylate several additional substrates, providing insight into the ever-increasing biological functions and roles MASTL plays in driving cancer progression and therapy resistance.

## Introduction

Microtubule-associated serine/threonine kinase-like (MASTL) is the human orthologue of Drosophila Greatwall (Gwl) kinase^1,2^, a protein essential for mitosis^3^. Since its discovery, MASTL has become known as a master regulator of mitotic entry and integrity^4–7^. More recently, its roles have expanded to include meiosis^8–10^, recovery from DNA damage^11–13^, DNA replication^14^, platelet maturation^15,16^, cell migration and actomyosin contraction^17^. Unsurprisingly, these roles have led to MASTL being identified as a potential oncogenic kinase^18^ with upregulation and over-expression correlating with poor patient outcomes in a variety of cancer types, including breast^19–21^, colon^22,23^ uterine^22^, head and neck^24^ and pancreatic cancer^25^. Conversely, knockdown of MASTL has also been shown to sensitize cancer cells to a variety of DNA damaging therapies including radiotherapy^26^, cisplatin^24,27^ and gemcitabine^25^. Understanding the functions of MASTL and how these might promote cancer development and therapy resistance are of considerable importance driving recent efforts towards the development of specific MASTL inhibitors^28–31^.

Currently the only validated substrate for MASTL is alpha-endosulfine (ENSA) and the highly related cAMP-regulated phosphoprotein 19 (Arpp-19). MASTL phosphorylates these proteins on a single conserved site (S-67 and S-62 respectively)^32,33^, converting ENSA/Arpp-19 into an unfair competitive inhibitor of the phosphatase PP2A-B55^34^. This inhibition is essential for correct mitotic entry and progression^1,35^, as it prevents PP2A-B55 from removing CDK1 phosphorylation events that drive correct mitotic entry and progression^36,37^. The MASTL/ENSA/PP2A axis is well conserved throughout evolution, going back as far as yeast *(S. cerevisiae),* where Rim15 (MASTL) phosphorylates the endosulfines Igo1 and Igo2, which then inhibit PP2ACdc55 (PP2A-B55)^38,39^. Interestingly, during nutrient starvation, Rim15 phosphorylates additional substrates including the transcription factors Msn2p/4p and Hsf1p^40^. This raises the possibility that there are also additional substrates beyond ENSA/Arpp-19 for MASTL, which might help explain the growing array of functions attributed to MASTL. Several recent efforts have been made to identify potential MASTL substrates with conflicting results. Work by Bisteau et al.^7^, identified 101 mitotic proteins that were dephosphorylated upon MASTL knockout in immortalized mouse embryonic fibroblasts (MEFs). Of these 6 were weakly phosphorylated in vitro by purified MASTL, but no decrease was observed using kinase dead MASTL, indicating that none are MASTL substrates^7^. However, work by Hermida et al.^41^ identified 56 potential substrates phosphorylated by MASTL using a Stable Isotope Labeled Kinase Assay-Linked Phosphoproteomics approach (siKALIP), suggesting that MASTL may phosphorylate a wide variety of targets. Consequently, additional research on the potential substrates of MASTL is needed.

Of note, MASTL is a member of the AGC kinase family that includes AKT and PKA, which each phosphorylate more than 130 and 370 substrates respectively^42^. A notable characteristic of the AGC kinase family is that its members often phosphorylate the same substrates and residues. This is in part due to a shared consensus motif for AGC kinases, with a strong preference for basic residues Arginine (R) and Lysine (K) N-terminal of a Serine/Threonine (S/T) phospho-site^42^. Currently, the consensus motif for MASTL substrates remains unknown, however, basic Lysine (K) residues are present N-terminal of the Serine phosphorylation site (-4 and -7 positions) in Arpp-19/ENSA, suggesting that MASTL may contain the same AGC family preference for basic residues.

Here we describe an unbiased whole cell lysate in vitro kinase screen combined with stable isotope labelling with amino acids in cell culture (SILAC) to identify novel substrates of MASTL kinase. We validated the utility of this approach using the related AGC family member AKT2, identifying known substrates and the preferred kinase motif for AKT2. Using active recombinant MASTL we demonstrated activity towards additional substrates using a peptide library array. Furthermore, using our SILAC based kinase screen, we identified 26 novel substrates potentially phosphorylated by MASTL along with the preferred amino acid residues. Subsequent in vitro kinase assays demonstrated that MASTL may phosphorylate YB1, TUBA1C and hnRNPM under certain conditions. Inhibition of MASTL with the small molecule inhibitor GKI-1, resulted in a small decrease in phosphorylation on S-633 of hnRNPM in HEK-293T cells. Collectively, these data suggest that MASTL may phosphorylate additional substrates, which likely support its expanding functional repertoire in cancer progression and therapy resistance.

## Results

### Validation of whole-cell lysate SILAC kinase screen using AKT kinase

**Figure 1:**
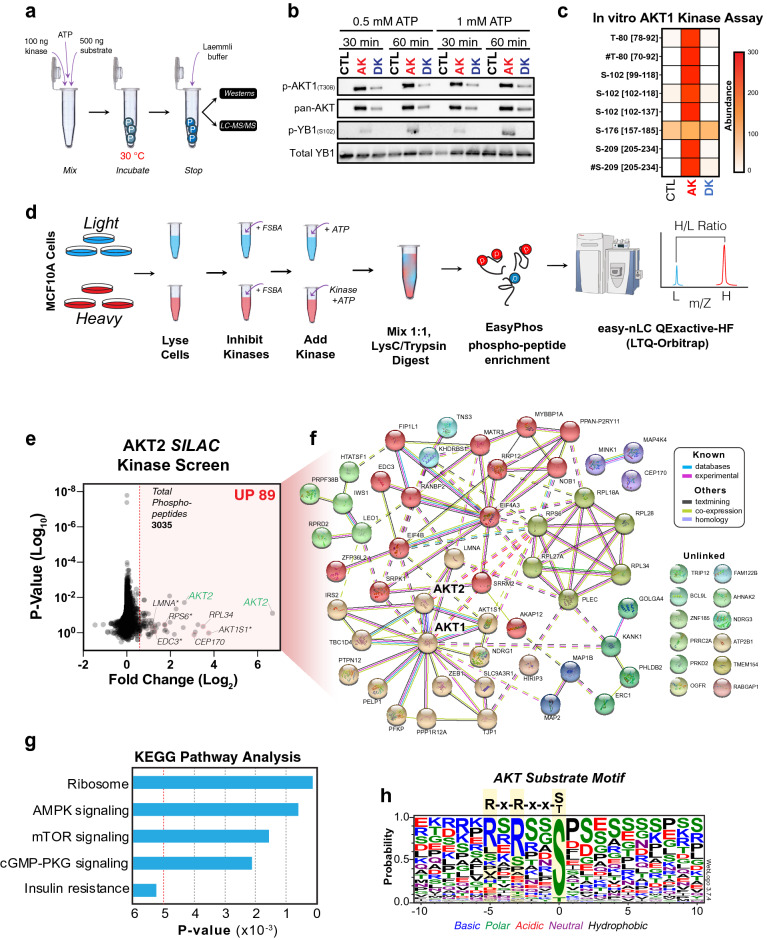
AKT SILAC kinase screen. **(a)** In-vitro kinase reaction steps between either no kinase (control, CTL), recombinant active kinase (active kinase, AK) or heat-treated kinase (denatured kinase, DK) with recombinant protein substrates. Up to four reaction conditions were tested (30 min and 60 min incubations, each in the presence of 0.5 mM ATP or 1 mM ATP). The resulting reaction mixtures were separated by SDS-PAGE and analyzed by western blot or further processed with in-gel digestion followed by liquid chromatography tandem mass spectrometry (LC–MS/MS). **(b)** The reaction between AKT1/YB1 was validated by immunoblot of known phospho-sites on active AKT1 (T-308), and the AKT1-mediated YB1 phospho-site (S-102). Pan-AKT was used to show the absence, presence and denaturing effect on active AKT-1, while Total YB1 was used to show the presence of YB1 substrate in each reaction. **(c)** Phospho-peptides identified by LC–MS/MS were analyzed by label-free quantitation. The abundance of phospho-peptides resulting from the AKT1/YB1 reaction (0.5 mM ATP, 60 min) are represented as a heatmap (mean of two independent reactions). Square brackets indicate peptide locations within YB1 on which each phosphorylation was found. Hashtags denote a co-modification within that peptide, e.g. carbamidomethylation, deamidation, and/or oxidation. **(d)** Graphical summary explaining the SILAC based whole cell lysate (in vitro) kinase assay. **(e)** Volcano plot of all identified phospho-peptides identified from the AKT2 SILAC kinase screen. A minimal Log_2_ Fold change cut-off < 0.5 (red shading) was taken as potential increased phosphorylation events. **(f)** STRING cluster analysis^49^ of proteins corresponding to increased phospho-peptides identified in E. **(g)** KEGG pathway analysis^50^ of proteins identified in F. **(h)** Phospho-peptides with a Log_2_ Fold change > 1.0 were aligned using WebLogo v3.7.4^52^. Residue colors indicate chemical properties of amino acids.

Identification of kinase-substrate relationships has been a major stumbling block that has hampered our understanding of these important cellular enzymes. To help overcome this, we utilized a modified version of the previously reported whole-cell lysate approach combined SILAC^43,44^. This assay attempts to reconstitute the cellular environment by mixing an active recombinant kinase with a concentrated cellular lysate that has had its endogenous kinases irreversibly inhibited, allowing phosphorylation events on any potential cellular substrate to be enriched and identified by mass spectrometry. To validate the assay, we took advantage of the well characterized and related AGC kinase family member AKT, for which active kinase is commercially available. First, in vitro kinase assays were performed (Fig. 1a) without (Control) or with active (AK) or heat-deactivated (DK) AKT1 kinase. These were incubated with purified recombinant YB1, a known AKT1 substrate^45,46^. Reactions were stopped after 30- or 60-min by the addition of Laemmli buffer, and samples were then processed for western blot and LC–MS/MS. Importantly, western blots confirmed that AKT1 was phosphorylated on T-308, indicating the kinase was active, while heat-deactivation (DK) markedly reduced the levels of phosphorylation (Fig. 1b). Furthermore, phosphorylation of YB1 on S-102, was only detected by western blot when incubated with active kinase (AK), with no bands detected in DK or control lanes (Fig. 1c). Notably, peak phosphorylation occurred after a 60 min incubation, while ATP concentration did not appreciably impact phosphorylation levels. Mass spectrometry confirmed these results and identified additional phosphorylation events on T-80 and S-209 of YB1 (Fig. 1c, Supplementary Table S1), which have not previously been associated with AKT1 activity. These results confirmed that commercially purchased active AKT kinase could successfully phosphorylate a known AKT substrate in vitro.

We therefore utilized active recombinant AKT2 kinase to validate the ability of the SILAC kinase screen to identify known and novel phosphorylation events. SILAC labelled MCF10A cells were cultured as previously described^19^. Briefly, both heavy and light labelled cells were lysed and treated with the pan-kinase inhibitor fluorosulphonylbenzoladenosine (FSBA). Lysates were concentrated and supplemented with ATP, with or without recombinant active AKT2 kinase (Fig. 1d). Following protease digestion, samples were phospho-enriched using the EasyPhos method^47^ and analyzed on a Q Exactive HF mass spectrometer.

The SILAC kinase screen identified a total of 3035 phospho-sites on 1085 proteins. Of these, 89 phospho-sites on 65 proteins (including AKT2) showed a Log_2_ fold-change greater than 0.5, indicating potential phosphorylation by AKT2 (Fig. 1e and Supplementary Table S3). Of note, approximately 30% of the high confidence phosphosites were proline directed, suggesting that there may be some contamination from another kinase. Within the 65 high confidence proteins, 9 have previously been reported as AKT substrates of which 8 are validated AKT phosphorylation sites^48^. These included AKT1S1 (T-246), EDC3 (S-161), RPS6 (S-236, S-240), LMNA (S-407), TBC1D4 (S-341, S-588), and IRS2 (S-306). STRING^49^ and KEGG pathway analysis^50^ found that 51 of the 65 proteins were directly or indirectly linked to AKT1/2 (Fig. 1f) and were strongly enriched for proteins involved in ribosome and AMPK and mTOR signaling (Fig. 1g), in line with the reported downstream targets of the AKT pathway^51^. WebLogo alignment^52^ of the phosphorylated peptide sequences centered around the phosphorylated residue, showed highly significant enrichment for serine phosphorylation with C-terminal enrichment of basic residues matching the published AKT phosphorylation motif (Fig. 1h). Of note, there were an additional 54 known protein substrates of AKT that fell below the Log_2_ fold-change significance threshold (Supplementary Table S2). Taken together, these results indicate that the in vitro kinase screen can identify known and potentially novel substrates along with the consensus phosphorylation motif for a well characterized AGC kinase family member.

### MASTL SILAC kinase screen

The above results indicated that our modified SILAC kinase screen could be used to identify novel substrates of a recombinant kinase. However, the screen requires high quality commercially available amounts of active MASTL kinase. We therefore partnered with Reaction Biology (previously ProQinase, Freiburg, Germany) to manufacture active human MASTL kinase. Briefly, GST-tagged full length wild-type human MASTL was expressed in Sf9 insect cells. Confluent cultures were treated with or without Okadaic Acid for 3 h and the soluble, active MASTL kinase purified by affinity chromatography (Supplementary Fig. S1a). The activity and ability of MASTL kinase to phosphorylate substrates was then determined using a S/T/Y-Physiological Kinase Substrate Finder Assay (Reaction Biology). Briefly, in vitro radioactive ATP (P^33^) based MASTL kinase assays were performed using a library of 720 peptides (13 amino acids long) derived from human protein sequences known to be phosphorylated in vivo. Over 150 peptides showed significant phosphorylation above autophosphorylation and background levels following incubation with active MASTL (Supplementary Fig. S1b,c, Supplementary Table S3). This included MBP (myelin basic protein), which has previously been used as an in vitro substrate for MASTL^53,54^ and EGFR and MYC, for which MASTL has been linked as an upstream regulator^23,25^. Taken together these results confirm that recombinant MASTL kinase possesses sufficient activity to be successfully used for in vitro assays. In addition, MASTL is capable of phosphorylating peptide sequences beyond ENSA/Arpp-19, under these in vitro conditions.

**Figure 2:**
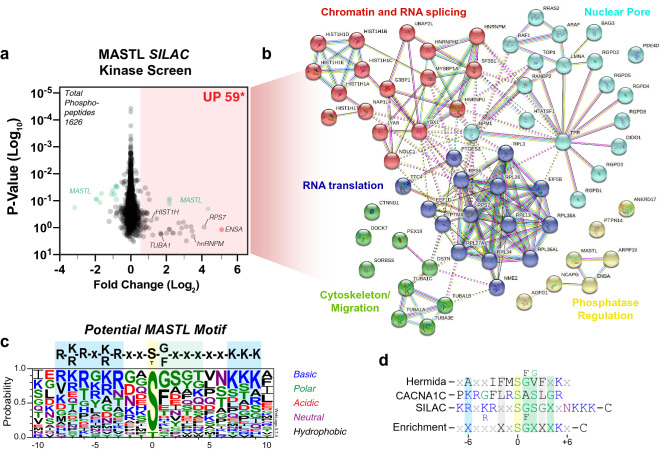
MASTL SILAC kinase screen. **(a)** Volcano plot of all identified phospho-peptides identified from the MASTL SILAC kinase screen. A minimal Log_2_ Fold change cut-off < 0.5 (red shading) was taken as potential increased phosphorylation events. **(b)** STRING^49^ cluster analysis of proteins corresponding to increased phospho-peptides identified in a. **(c)** Phospho-peptides with a Log_2_ Fold change > 1.0 were aligned using WebLogo v3.7.4^52^. Residue colors indicate chemical properties of amino acids. **(d)** Alignment of top phosphorylated peptide (CACNA1C) from the S/T/Y Physiological Kinase Substrate Finder Assay (Reaction Biology), with the potential MASTL motif identified in c.

Therefore, we next repeated the SILAC kinase screen (Fig. 1d), substituting MASTL for AKT2. MCF10A cell lysates were again used as they express low levels of endogenous MASTL^19^. A total of 1626 phospho-peptides were identified with 59 peptides showing a Log_2_ fold increase greater than 0.5 (Fig. 2a, Supplementary Table S4). These 59 phospho-peptides mapped to 67 potential proteins, including exogenous active MASTL (Fig. 2b). The higher number of proteins was due to the homology of some peptides matching with multiple closely related protein variants (e.g., HIST1H1E, HIST1H1A, HIST1H1T etc.). Importantly, the top phospho-site identified was S-62/67 on ENSA/Arpp-19, providing a positive internal control for the SILAC assay. STRING analysis^49^ of all potential phospho-peptides showed strong enrichment for proteins involved in regulation of mRNA translation, splicing, nuclear pore function cytoskeleton/migration and the expected PP2A phosphatase regulation. Alignment of phosphorylated peptide sequences, which showed a > 1.0 Log_2_ fold increase, revealed an enrichment for serine phosphorylation with C-terminal preference for basic residues (K, R) between the -3, to -7 positions, along with N-terminal polar residues from + 1 to + 4 and basic (K) residues from + 7 to + 9 (Fig. 2c). Notably, the top phosphorylated peptide (CACNA1C) from the S/T/Y-Physiological Kinase Substrate Finder Assay, showed some similarity to the MASTL consensus motif with preference for basic residues at the -6 position and polar residues from + 1 to + 4 (Fig. 2d). In support, Hermida et al.also identified a strong enrichment of polar and hydrophobic residues at the + 1 to + 4 positions and a basic residue at + 5^41^. Taken together, these data indicate a potential MASTL motif with a basic residue at the −6 and + 5 positions, hydrophobic residues at −2, and polar/hydrophobic residues from + 1 to + 4. In summary, the SILAC Kinase screen results demonstrate that MASTL may phosphorylate several potential novel substrates and defines possible kinase motif for MASTL.

### In vitro kinase validation of MASTL substrates

**Figure 3:**
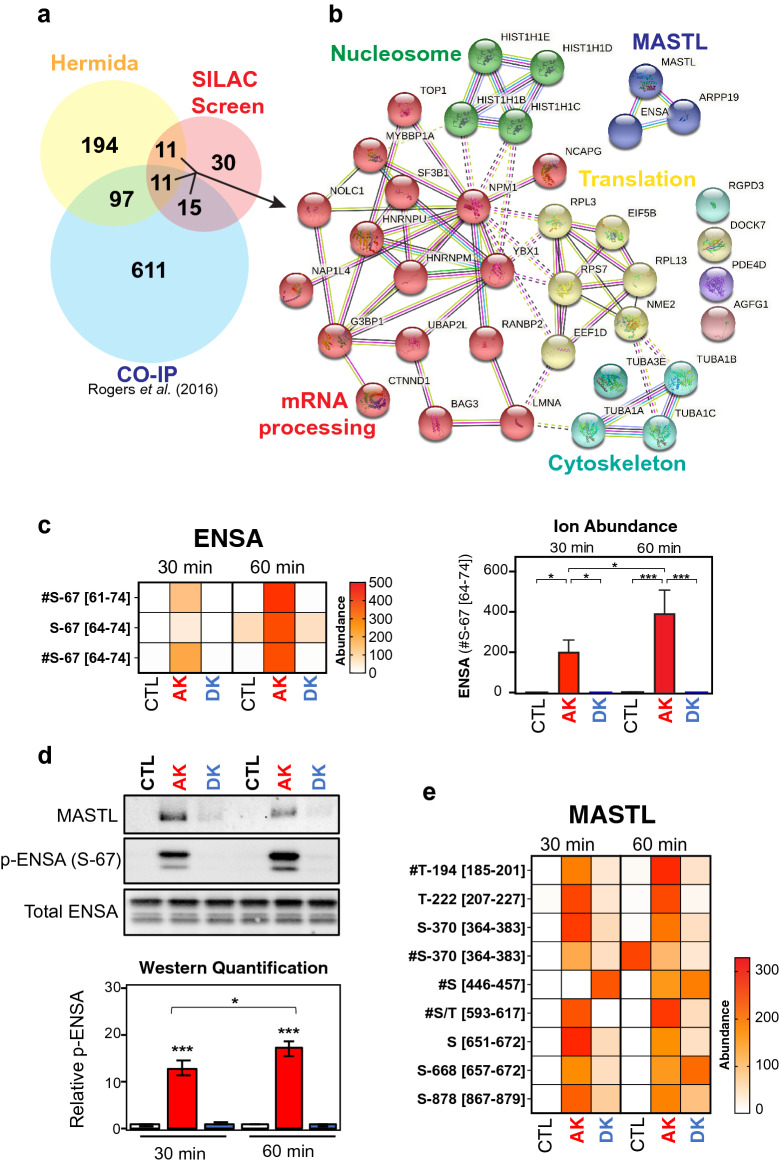
In vitro kinase assay validation of ENSA phosphorylation by MASTL. **(a)** Venn diagram comparing previously identified MASTL interacting proteins by co-immunoprecipitation^55^ with those identified from A, and **(b)** subsequent STRING cluster analysis^49^ of common proteins. **(c)** The abundance of each ENSA phospho-site activated in reactions between MASTL kinase and ENSA. The ion abundance for the #Ser67 [64–71] phospho-peptide is normalized to the 60 min control (CTL) reaction. Error bars display the standard error of the mean from three independent reactions. Square brackets indicate peptide locations within the protein on which each phospho-site was found. Hashtags denote a co-modification within that peptide, e.g., carbamidomethylation, deamidation, and/or oxidation (detailed in Supplementary Table S6). **(d)** Western blot confirmation of ENSA phosphorylation at S-67 in the presence of active MASTL kinase. Total ENSA was used as the loading control and normalization factor for densitometry analysis. P-ENSA/Total was further normalized to the 60 min control (CTL) reaction. Error bars display the standard error of the mean from three independent reactions. **(e)** The abundance of MASTL phospho-sites present in reactions with ENSA are represented here as a heat map. Two reaction conditions were tested (60 min incubation in presence of 0.5 mM ATP or 1 mM ATP). Phospho-site abundances were calculated using label-free quantitation.

Our next goal was to functionally characterize the candidate substrate proteins from the SILAC screen. To further refine the list of potential substrates, we cross-referenced the phosphorylated proteins with our previously published list of proteins that co-immunoprecipitated (Co-IP) with MASTL from mitotic HeLa cell extracts^55^ and MASTL substrates predicted by Hermida et al.^41^. There were 11 proteins that were present in all three datasets, an additional 11 common to our SILAC screen and Hermida’s dataset, and a further 15 common to SILAC screen and our previous Co-IP (Fig. 3a, Supplementary Table S4). These 3 groups of 37 proteins were clustered around the regulation of cell migration, RNA splicing, RNA translation and chromatin condensation (Fig. 3b). We selected 6 proteins spanning these 3 groups (LMNA, YBX1, TUBA1C, NPM1, HNRNPM and ENSA) and 2 found only in our SILAC screen (LYRA, RPS6), for further validation by in vitro kinase assay. These were selected based on previously reported functions that correlated with any of the known roles for MASTL (e.g., mitosis, EMT, cancer), the availability of commercial recombinant purified protein and those with the highest Log_2_ fold-change from our SILAC screen. Before beginning more detailed functional analysis we confirmed the activity of MASTL towards ENSA under the same in vitro assay conditions used for AKT1 (Fig. 1a). We compared ENSA phospho-peptide intensities between ATP-only, active MASTL and heat-deactivated MASTL at different timepoints as a marker for specific kinase activity. Significant phosphorylation of ENSA on S-67 was only detected in presence of active kinase (AK), with peak phosphorylation observed in the 60 min reaction (Fig. 3c). Western blot analysis confirmed that active MASTL kinase (AK), significantly and specifically phosphorylated ENSA on S-67, with a 60 min incubation showing consistently higher levels of phosphorylation (Fig. 3d). Interestingly, multiple phospho-peptides from MASTL were also detectable in our mass spectrometry analysis. Of these, phosphorylation on T-194, which is essential for MASTL activity^54^, was significantly more abundant in the active kinase sample compared to control, and importantly heat deactivation of MASTL (DK) reduced the abundance of T-194 and other phospho-sites (Fig. 3e). This, combined with specific phosphorylation on ENSA, confirmed that recombinant MASTL possessed sufficient activity under in vitro assay conditions.

**Figure 4:**
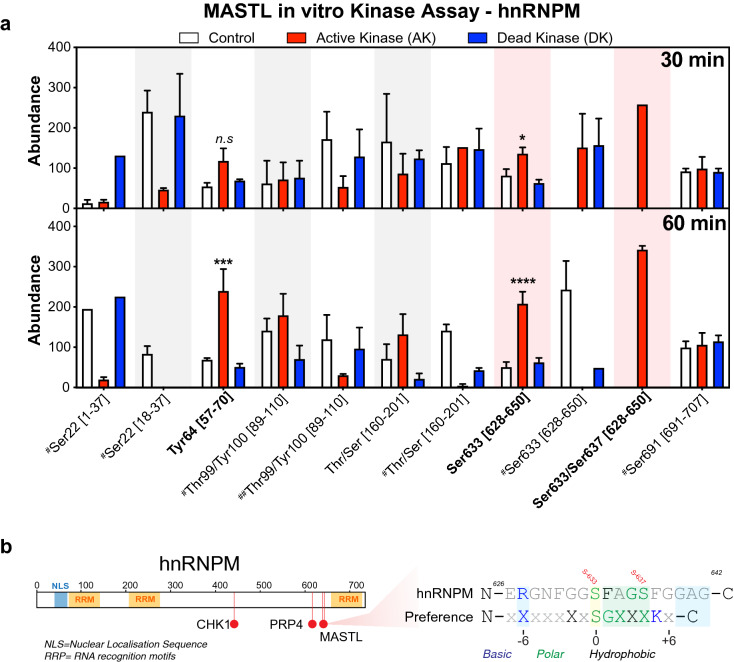
In vitro kinase assay validation of hnRNPM phosphorylation by MASTL. (**a**) The ion abundance of each phospho-site activated in triplicate reactions between MASTL kinase and hnRNPM (as detailed in this figure legend). Phospho-site abundances were calculated using label-free quantitation. Square brackets indicate peptide locations within the protein on which each phospho-site is found. Single hashtags denote a co-modification within that peptide, e.g., carbamidomethylation, deamidation, and/or oxidation. Double hashtags denote a different combination of co-modifications within the same peptide (detailed in Supplementary Table S7). Error bars display the standard error of the mean from three (n = 3) independent reactions. **(b)** Schematic of hnRNPM showing nuclear localisation domain (NLS) RNA recognition motifs (RRM) and phospho-sites currently annotated in PhosphoSitePlus (Chk1 and PRP4) along with the novel MASTL sites identified here. Sequence alignments of the MASTL phosphorylation sites within hnRNPM (S-633 and S-637), compared to validated substrates Arpp19/ENSA and the possible MASTL motif identified from our SILAC screen (Fig. 2d).

The seven remaining substrates were subjected to a single-shot in vitro kinase assay at 0.5- and 1.0-mM ATP for 60 min, and where sufficient substrate was available, 30 min reactions were also performed (Supplementary Fig. S2, Supplementary Table S5). Strong and specific phosphorylation of ENSA was again observed (Supplementary Fig. S2), however no phosphorylation of LYRA was seen in any reaction. Similarly, while PTGES3 (p23) was phosphorylated, this was not specific, with phosphorylated residues detected under control (CTL) and dead kinase conditions (DK), indicating that neither LYRA nor p23 are likely substrates of MASTL. In contrast, a slight increase in phosphorylation in the presence of the active kinase (AK) was observed under some conditions for RPL36A and RPS6, while for hnRNPM, TUBA1C and YB1 more prominent increases in AK samples were present under multiple conditions (Supplementary Fig. S2, blue rectangles). Notably, phosphorylation of hnRNPM on S-633 and S-48 on TUBA1C were also identified in the MASTL SILAC screen (Fig. 2). While phosphorylation of YB1 on S-209 showed the strongest specificity, this site was not seen in our MASTL SILAC screen, but was found in our AKT1 SILAC screen (Fig. 1e). Based on these results, we selected hnRNPM as a potential novel substrate for further validation using more detailed in vitro kinase assays. Triplicate assays identified 13 unique phospho-peptides that were present in at least two out of three replicates. Increased phosphorylation of Y-64 was observed at both 30 and 60 min; however, this is likely non-specific as MASTL is a serine/threonine directed kinase and may indicate contamination with another kinase. Of the remaining sites only S-633/637 was significantly and specifically more abundant in the active kinase (AK) reaction (Fig. 4a, Supplementary Table S6). Interestingly, S-633 is located just downstream from the C-terminal RNA recognition motif (RRM) of hnRNPM (Fig. 4b) and shows some homology with the potential MASTL motif (Fig. 4b), with hydrophobic/polar residues enriched in + 1 to + 4 positions, and a basic residue at -6. To assess this further, we over-expressed GFP-tagged full-length hnRNPM in asynchronous HEK-293 T cells that were then enriched in G2/M using Paclitaxel or not (CTL), and then treated with or without the MASTL inhibitor GKI-1 for 8 h (Supplementary Fig. S3a). Western blot analysis confirmed that Paclitaxel successfully enriched cells in G2/M, with increased phosphorylation on S-67 of ENSA (p-ENSA) and S-10 of Histone H3 (p-Histone H3). Inhibition of MASTL with GKI-1 reduced p-ENSA phosphorylation in both untransfected controls (CTL) and GFP-wt-hnRNPM transfected G2/M samples by approximately 30% (Supplementary Fig. S3b). We next isolated over-expressed hnRNPM by immunoprecipitation using GFP specific antibodies with subsequent peptide purification by PAGE in-gel digestion (Supplementary Fig. S3c,d). Mass spectrometry analysis of the excised bands revealed 9 phosphorylated residues on hnRNPM (Supplementary Fig. S3e, Supplementary Table S7). Analysis of S-633 phosphorylation revealed a noticeable decrease in phosphorylation in the G2/M enriched compared to the asynchronous samples, which was not observed for S-86. Treatment with GKI-1 resulted in weak reduction in phosphorylation in interphase on S-86 (~ 8.5%) and S-633 (~ 3%), while in G2/M enriched samples MASTL inhibition further reduced S-633 phosphorylation (~ 10.5%), while neighboring S-618 increased by ~ 4.5% and S-86 was reduced by ~ 4.5% (Supplementary Fig. S3f). The mild reductions observed with GKI-1 may be partly due to slow dephosphorylation kinetics for S-633 under these conditions and or to sub-optimal inhibition of MASTL, as noted by only a ~ 30% reduction on ENSA S-67 phosphorylation (Supplementary Fig. S3b). Identifying, where and at what stage of cell division hnRNPM is phosphorylated on S-633 will be critical for future validation experiments. Taken together, these results indicate that MASTL is capable of phosphorylating hnRNPM under certain in vitro conditions, while inhibition of MASTL with GKI-1 may slightly reduce hnRNPM phosphorylation on S-633 in HEK-293 T cells.

## Discussion

Here we have presented a broad analysis of MASTL substrates using a reconstituted and adapted cellular protein environment for use as a novel discovery tool. Our SILAC kinase screen approach successfully identified known and novel substrates for AKT2 and MASTL kinase. One notable limitation we observed with the AKT SILAC screen was the possible contamination from other kinases that may co-purify with the recombinant AKT2 kinase. This may also be a potential confounding issue with the MASTL SILAC screen. In addition, use of fivefold excess MASTL for the in vitro kinase assays may have produced some false-positive results. Consequently, use of an additional kinase dead mutant control and additional in vitro assay conditions that use lower concentrations of kinase will be necessary for any future validation experiments. Despite these limitations, our SILAC and in vitro assays both correctly identify known substrates and their phosphorylation sites for MASTL and AKT2, indicating that the approach can work. Cross referencing our SILAC kinase assay data with our previous co-immunoprecipitation results and those of Hermida^41^ suggests that hnRNPM and to a lesser extent TUBA1C and YB-1 may be MASTL substrates.

Tubulin (TUBA1C) has previously been implicated as a potential MASTL substrate using a siKALIP approach^41^. In support, we have also shown that it can associate with microtubules during mitosis, decorating the mitotic spindle^1^, and co-precipitates with tubulin from mitotic cell extracts^19^. Combined with our observation here that under certain in vitro conditions MASTL can phosphorylate the core microtubule protein α-tubulin (TUBA1C) on multiple sites (T-41, S-48, and T-292), suggests that MASTL may regulate microtubule function. Notably, knockdown of MASTL results in multiple mitotic spindle and chromosome alignment defects^1,2^. Of note, the related AGC kinase, PKC, phosphorylates α-tubulin on S-165, stimulating microtubule dynamics^56,57^, supporting the potential of AGC kinases as regulators of α-tubulin. Consequently, our results raise the possibility that MASTL could regulate spindle assembly by both maintaining CDK1 substrate phosphorylation, and by direct regulation of tubulin dynamics.

Our previous work identified several links between MASTL and epithelial mesenchymal transition (EMT), with MASTL over-expression overriding contact inhibition and disrupting cell migration in MCF10A breast epithelial cells^19^. This corresponded with increased phosphorylation of several members of the β-catenin pathway and RPS6. Interestingly, hnRNPM has been associated with increased activation of β-catenin signaling in breast cancer^58^, and increased EMT in breast^59^ and prostate cancer^60^. Both our results here and that of Hermida et al.^41^ identified the same S-633 phosphorylation site on hnRNPM, as a potential site phosphorylated by MASTL, while inhibition of MASTL with GKI-1 resulted in a possible reduction in S-633 phosphorylation in HEK-293 T cells. Notably, the S-633 site on hnRNPM has been found in breast cancer and ischemic tumor tissues by mass spectrometry^61,62^, suggesting that this site is phosphorylated under certain conditions in vivo. However, no upstream kinase has been assigned for this phosphorylation site, nor has any functional significance been attributed. Of note, the phosphorylation site is located just next to the C-terminal RNA recognition motif, hence phosphorylation of S-633 could potentially regulate the binding of target RNA sequences, hnRNPM’s splicing activity, or impact binding of upstream regulatory proteins, such as AKAP8^59^. Elucidating the links between hnRNPM and MASTL may give a greater understanding to the role MASTL plays in regulating EMT in breast cancer.

MASTL over-expression has also been linked with hyper-activation of the PI3K/AKT/mTOR pathway in humans^63^, a function that may be related to its role in regulating nutrient response in yeast^40,64^. Interestingly, RPS6 plays an important role in regulating nutrient-mTORC1 signaling in human cells and is phosphorylated by multiple AGC kinases including AKT and p90RSK^63,65^. Similarly, we found that YB1, which also plays several key roles in the AKT pathway^46^, could be phosphorylated by both AKT1 and MASTL on S-209 in our SILAC screen and in vitro assays and immunoprecipitates with MASTL^55^, suggesting this site may be phosphorylated by multiple AGC family members including MASTL. It will be of future interest to determine if phosphorylation by MASTL on either RPS6 and YB1 plays any role in their regulation and if this impacts nutrient response within mammalian cells.

In summary, here we show that under certain in vitro conditions MASTL has the potential to act like other AGC kinases and phosphorylate several substrates in addition to ENSA/Arpp19. Many of these new potential substrates, such as tubulin and hnRNPM, may help explain the growing list of MASTL functions including how MASTL promotes cancer, EMT and chemoresistance. Given MASTL’s rising role in cancer, there has been growing interest in the development of specific MASTL inhibitors^28,30,31^. We hope that our characterization of potential cellular targets and identification of a possible motif for MASTL will aid their development and help improve next-generation inhibitors. Consequently, it will be of critical importance to further validate our SILAC method using alternative screening approaches^66^ and confirm the physiological importance of these novel MASTL substrates with additional controls and functional assays in future research.

## Materials and methods

A full list of reagents used along with catalogue number and suppliers can be found in Supplementary Table S8.

### Cell lines

MCF-10A breast cells were purchased from Sigma (#CLL1040) (38). MCF-10A cells were grown in DMEM/F12 (1:1) supplemented with 5% horse serum, 20 ng/mL recombinant human EGF, 0.5 μg/mL hydrocortisone, 100 ng/mL cholera toxin, and 10 ug/mL bovine insulin as previously described^19^. HEK-293 T cells (ATCC CRL-11268) were grown in Advanced DMEM supplemented with 5% fetal bovine serum and 1% GlutaMAX. All cell lines were tested regularly for mycoplasma using DAPI staining.

### Proteins, antibodies and reagents

Active MASTL kinase (#1593-0000-2) and the Ser/Thr/Tyr-Physiological Kinase Substrate Finder Assay Service was purchased from and performed by Reaction Biology (previously ProQinase, Freiburg, Germany). Purified recombinant AKT2 was purchased from Active Motif (#81146). Purified recombinant active AKT1 (#ab62279) and recombinant proteins ENSA (#ab92932), YB1 (#ab187443), hnRNPM (#ab226407), p23 (#ab113183), LYAR (#ab163160), RPL36A (#ab159415), TUBA1C (#ab164660) were purchased from Abcam, and RPS6 (#H00006194-P01) was purchased from Abnova. Kinase reaction reagents included Tris-base, hydrochloric acid, magnesium chloride hexahydrate, DL-dithiothreitol and were purchased from Sigma, and adenosine 5’-triphosphate from New England Biolabs. Antibodies used for western blotting included anti-MASTL, phospho-YB1 (S-102), pan-AKT, phospho-AKT1 (T-308), phospho- Arpp-19/ENSA (S-62/67), and phospho-histone H3 (Cell Signaling Technology), as well as anti-YB1, anti-rabbit IgG HRP and anti-mouse IgG HRP (Abcam), and anti-His-ENSA^14^. GFP antibody validated for immunoprecipitation (IP) was from Cell Signaling Technology. IP reagents included Pierce IP lysis buffer and Protein G magnetic beads (Thermo Scientific). Reagents for mass spectrometry sample preparation including LC–MS grade water and formic acid were purchased from Pierce, HPLC grade trifluoroacetic acid and LiChrosolv acetonitrile from Sigma.

### Whole cell lysate SILAC kinase screen: sample preparation

MCF-10A cells were labelled by stable isotope labelling of amino acids in cell culture (SILAC) as previously described^19^. Briefly, cells were cultured for six population doublings in SILAC F12:DMEM (1:1) (Thermo Scientific) supplemented with either “heavy” lysine-^13^C_6_^14^N_2_ (Lys8) and arginine-^13^C_6_^14^N_4_ (Arg10), or the respective “light” counterparts (Lys0, Arg0). Labelled amino acid incorporation was checked routinely. Protein was harvested by scraping in 1 mL of ice-cold NP-40 buffer (1% NP-40, 150 mM NaCl, 50 mM Tris HCl pH 7.4, 10% Glycerol) plus protease inhibitor cocktail (Sigma) and PhosStop (Roche) and stored at -80 °C. 100 mM 5'-4-fluorosulphonylbenzoyladenosine (FSBA) in DMSO was slowly diluted 1:5 in pre-warmed NP-40 buffer at 37 °C. To irreversibly inhibit endogenous kinase activity, 1 mL of the FSBA solution was added to thawed lysates and shaken at 30 °C for 30 min at 1200 rpm. Precipitated FSBA was removed by centrifugation and the supernatant was concentrated to 2.5 mL in a 10,000 MW centrifugal filter (Amicon). 1 mg of concentrated lysate was diluted 1:1 in 2 × kinase assay buffer (50 mM Tris HCl pH 7.4, 10 mM MgCl_2_, 3 mM MnCl_2_, 3 µM Na_3_VO_4_, 2 mM DTT, and 500 µM ATP). 50 µg of recombinant purified MASTL kinase (Reaction Biology) or 10 µg AKT2 (Active Motif) was added to the heavy sample and incubated at 32 °C for 3 h under gentle agitation. The reaction was stopped by adding GdmCl buffer (6 M GdmCl, 100 mM Tris pH 8.5, 20 mM TCEP, 100 mM IAM) to each sample. Kinase reactions were heated to 95 °C for 5 min, cooled on ice, then kept at RT in the dark for 30 min to allow for peptide reduction and alkylation. Heavy and light samples were mixed 1:1 and 4 × volumes of cold acetone were added to each mix and incubated at −20 °C overnight. Precipitated proteins were digested in Lys-C, and trypsin at 100:1 (w:w) overnight at 37 °C. The resultant mixture was then enriched for phosphorylated peptides using the EasyPhos method as described^47^. Briefly, phospho-peptides were bound to TiO_2_ beads (5 µm Titansphere, GL Sciences) at a ratio of 10:1 (protein:beads) at 40 °C for 5 min. After washing (80% ACN, 0.5% 5% trifluoroacetic acid), phospho-peptides were eluted (40% ACN, 15% Ammonium Hydroxide), dried, and loaded onto in-house packed dual layer SDB-RPS stage tips (3 M empore). The stage-tips were desalted and phospho-peptides eluted (80% ACN, 5% ammonium hydroxide), dried and stored at −80 °C until mass spectrometry analysis.

### Whole cell lysate SILAC kinase screen: mass spectrometry and data analysis

Digested phospho-peptides were analyzed as previously described. Briefly, a 50 cm × 75 μm fused silica column, packed in-house using 1.9 μM C18AQ particles using an Easy nLC-1200. Peptides were separated using a 195 min gradient using a binary buffer system of Buffer A (0.1% formic acid) and Buffer B (80% ACN, 0.1% formic acid) at a flow rate of 300 nL/min. Peptides were eluted with a gradient of 5–30% buffer B over 150 min, followed by 30–60% buffer B over 5 min, and 60–95% buffer B over 5 min. Peptides were analyzed on a Q-Exactive HF mass spectrometer operated in positive-ion DDA mode, with one full scan (300–1650 *m/z*, *R* = 35,000 at 200 *m/z*), at a target of 3e^6^, selecting top 20 most abundant precursor ions (isolation window = 1.4 *m/z*, ion target 3e^5^) for HCD fragmentation (NCE = 27%) and MS2 scan (*R* = 35,000 at 200 *m/z*). Thermo RAW files were generated in centroid mode and analyzed using MaxQuant (v.1.5.3.30) with integrated Andromeda search engine, searching against the human whole proteome, with additions (Uniprot release 07.2018; 21,050 entries) Default MaxQuant settings were used, with 1% peptide spectral match (PSM) false discovery rate (FDR) followed by further filtering to 1% protein FDR. Precursor mass tolerance was set to 20 ppm for a first search followed by mass recalibration and 7 ppm tolerance for second search. Fragment ion tolerance was set to 7 ppm. The searches were conducted with carbamidomethylation of Cys set as a fixed modification. Phosphorylation of Serine, Threonine and Tyrosine, oxidation of Met and “light” Lysine and Arginine were replaced by “heavy” isotope-labelled amino acids 13C615N4-l-Arginine (Arg 10) and 13C615N2-l-Lysine (Lys 8) with Arginine and Lysine set as variable modifications. The match between runs and re-quantify options were enabled with matching elution time of 1.0 min. Bioinformatics were performed in Perseus (1.5), and Microsoft Excel (2010). Briefly, SILAC H/L ratios determined by MaxQuant were inverted for label-swap experiments, and Log_2_ transformed prior to statistical analysis. Non-significant Log_2_H/L ratios were eliminated using the Student’s t-test with Benjamini–Hochberg adjustment for multiple comparisons, with P < 0.05 as a significance cut off, ratios > 0.5 were considered significantly altered. KEGG pathway enrichments^50^ were determined from the parent protein identified from the individual phospho-peptides^64^and interaction networks were produced using STRING v11.5^49^.

### In vitro kinase assays

In vitro kinase assay reaction mixtures (40 μl total) contained 500 ng of AKT1 or MASTL kinase and 100 ng of substrate protein in kinase reaction buffer (50 mM Tris–HCl, 10 mM MgCl_2_, 2 mM dithiothreitol, pH 7.4) in the presence of 0.5 mM or 1 mM ATP. Reactions were performed at 30 °C for 30 min and 60 min, then quenched with 2× SDS–polyacrylamide gel electrophoresis sample buffer (Bolt, Invitrogen).

### GFP-wt-hnRNPM overexpression in HEK-293 T cells and immunoprecipitation

HEK-293 T cells (ATCC CRL-11268) were grown in six-well plates and treated for 8 h with and without 10 nM paclitaxel (Cayman Chemical) and 50 μM GKI-1 (MASTL inhibitor; MedChemExpress). Cells were lysed directly into SDS-PAGE reducing sample buffer and used for western blot confirmation of paclitaxel-induced phosphorylation of histone H3 and ENSA—indicating arrest at G2/M, as well as GKI-1-induced inhibition of these phospho-proteins—indicating MASTL kinase inhibition. HEK-293 T cells were then seeded in 10 cm culture dishes or 6-well plates and grown to 70% confluency before transfection with GFP-wt-hnRNPM using Lipofectamine 3000 (Invitrogen). Cells were treated 16 h later with and without paclitaxel and GKI-1 as before. At 8 h post-treatment, cells were harvested with IP lysis buffer containing Halt protease and phosphatase inhibitor cocktail (Thermo Scientific). Protein concentration was measured using a BCA kit (Thermo Scientific). Immunoprecipitation was then carried out with 500 ug protein and anti-GFP antibody (1:50 dilution), overnight at 4 °C, followed by pull-down with prewashed Pierce Protein G magnetic beads (Thermo Scientific) for 1 h at room temperature, before collecting with SDS-PAGE reducing sample buffer. Samples were also set aside for western blot confirmation of input, flow-through and IP fractions using anti-hnRNPM. Pulled-down fractions then underwent gel electrophoresis and prepared for mass spectrometry as described below.

### SDS-PAGE and western blotting

Kinase reaction samples were heated for 10 min at 70 °C, then cooled on ice for 2 min. The full sample volume (less a small amount used for western blotting) was loaded onto Bolt 4–12% Bis–Tris Plus gels and run under reducing conditions with Bolt MES Running Buffer (Invitrogen) at 180–200 V. Prestained molecular weight markers were also included (SeeBlue Plus2, Invitrogen; GangNam-STAIN, iNtRON Biotechnology). Once kinase and substrate molecular weights were sufficiently separated, gels were washed and stained according to manufacturer’s instructions using SimplyBlue SafeStain (Invitrogen) before proceeding to in-gel digestion and mass spectrometry. For western blotting, gels were washed in water and proteins transferred onto PVDF membranes using the iBlot2 system (Invitrogen). Membranes to be probed for phospho-proteins were blocked in 5% bovine serum albumin (BSA; Sigma) in tris-buffered saline (TBS) containing 0.1% Tween-20 (TBS-T), or otherwise in 5% low fat milk in TBS-T. Primary antibodies were diluted in 3% BSA/TBS-T and incubation was carried out overnight at 4 °C. Secondary antibodies were diluted in 5% LFM/TBS-T or 3% BSA/TBS-T (for phospho-proteins) and incubation was carried out for 1–2 h at room temperature. All membrane washes were carried out with TBS-T, except for the final wash before imaging, which used TBS. Protein bands were detected by chemiluminescence using Clarity Western ECL Substrate (Bio-Rad) and the ChemiDoc imaging system (Bio-Rad). All original, un-cropped blots are found in Supplementary Fig. S4. Densitometry was analyzed with ImageLab software.

### In-gel digestion

Stained protein bands were excised with a clean scalpel and underwent destaining, reduction-alkylation and digestion using an In-Gel Tryptic Digestion Kit (Thermo Scientific). Manufacturer’s instructions were followed. Briefly, one volume of reagent was used per protein band, and trypsin digestion was carried out on a heat block overnight (16–18 h) at 37 °C.

### Mass spectrometry of in vitro kinase assay and immunoprecipitated samples

Digested samples were desalted, purified, and concentrated using C18 ZipTips (Z720070 (discontinued), Millipore), then dried by vacuum centrifugation and reconstituted in loading buffer (3% acetonitrile, 0.1% formic acid). Samples were injected onto a 50 cm × 75 μm internal diameter column (packed in-house with 1.9 μm Pur 120 C18 particles) using a Thermo Ultimate-3000 UHPLC. Peptides were separated using a 110 min gradient with a binary buffer system (buffer A = 0.1% formic acid; buffer B = 80% acetonitrile, 0.1% formic acid) at a constant flow rate of 0.250 μl/min. Peptides were eluted with a gradient of 5% buffer B over 30 min, then 5–10% buffer B over 1 min, then 10–35% buffer B over 59 min, then 35–95% buffer B over 3 min, then maintained at 95% buffer B for 4 min, then 95–5% buffer B over 1 min, then 5% buffer B maintained for a further 12 min. The column was maintained at 30 °C. Data-dependent acquisition (DDA) was performed with a Q Exactive Plus Orbitrap mass spectrometer operating in positive-ion mode, with a full-scan MS1 measured at 35,000 resolution (350 to 1550 *m/z* scan range; 50 ms injection time; 3 × 10^6^ automated gain control (AGC) target) followed by isolation of up to 20 most abundant precursor ions for MS/MS (1.2 *m/z* isolation; 26 normalized collision energy; 17,500 resolution; 60 ms injection time; 3 × 10^5^ AGC target). Thermo RAW files were generated in centroid mode. Raw data were processed with Proteome Discoverer v2.3 and v2.4 and searched with Sequest HT against the human UniProt database (07.2018; 21,050 entries). Full trypsin was set as the enzyme used with a maximum of two missed cleavages permitted. The mass tolerance for precursor ions was set to 10 ppm. The searches were conducted with carbamidomethylation on cysteine set as a static modification. Carbamidomethylation on histidine and lysine, phosphorylation of serine, threonine and tyrosine, deamidation of asparagine and glutamine, and oxidation of methionine were set as variable modifications. An automatic target FDR of 1–5% was set. Relative abundance levels of phosphorylation were found with label-free quantitation (LFQ) using the Minora Feature Detector.

## Experimental design and statistical rationale

For SILAC kinase screening, two independent (n = 2) biological replicates were performed. Average Log_2_H/L ratios from the two replicates greater than > 0.5 were taken as potentially increased (phosphorylated) substrates for AKT and MASTL. Further stringency was applied using a Student’s t-test, with P < 0.05 taken as significant. For MASTL, substrates were further refined by cross-referencing with previously identified proteins that co-immunoprecipitated with MASTL from mitotic HeLa cell extracts^55^. For the AKT1 + YB1 in vitro kinase assay, two (n = 2) biological replicates were performed, and for MASTL + ENSA and MASTL + hnRNPM in vitro kinase assays, three (n = 3) independent biological replicates were performed. Statistical significance for western blot was determined using two-way ANOVA with Tukey’s multiple comparison’s test, and significance for mass spectrometry results was determined using an unpaired one-way ANOVA with Tukey’s multiple comparisons test using GraphPad Prism software (v9.1.0).

## Supplementary Information

Supplementary Figure S1.

Supplementary Figure S2.

Supplementary Figure S3.

Supplementary Figure S4.

Supplementary Table S1.

Supplementary Table S2.

Supplementary Table S3.

Supplementary Table S4.

Supplementary Table S5.

Supplementary Table S6.

Supplementary Table S7.

Supplementary Table S8.

Supplementary Legends.

## Data Availability

All raw mass spectrometry data files, and MaxQuant and Proteome Discoverer output files have been deposited to the ProteomeXchange Consortium via the PRIDE partner repository^67^ with the identifier PXD028678 (http://proteomecentral.proteomexchange.org).

## Abbreviations

Arpp-19: CAMP-regulated phosphoprotein 19
CDK1: Cyclin dependent kinase 1
EMT: Epithelial mesenchymal transition
ENSA: Alpha-endosulfine
FSBA: 5'-4-Fluorosulphonylbenzoyladenosine
hnRNPM: Heterogeneous nuclear ribonucleoprotein M
IAM: Iodoacetamide
K: Lysine
MASTL: Microtubule-associated serine/threonine kinase-like
PP2A: Protein phosphatase 2A
PKA: Protein kinase A
PKC: Protein kinase C
R: Arginine
siKALIP: Stable isotope labeled kinase assay-linked phosphoproteomics
SILAC: Stable isotope labelling with amino acids in cell culture
S/T/Y: Serine/threonine/tyrosine
TCEP-HCl: Tris(2-carboxyethyl)phosphine hydrochloride
