# Supplementary figures and images for: SILAC kinase screen identifies potential MASTL substrates
Source: Sci Rep. 2022 Jun 22;12:10568. doi: 10.1038/s41598-022-14933-0 (PMC9217955; doi:10.1038/s41598-022-14933-0)

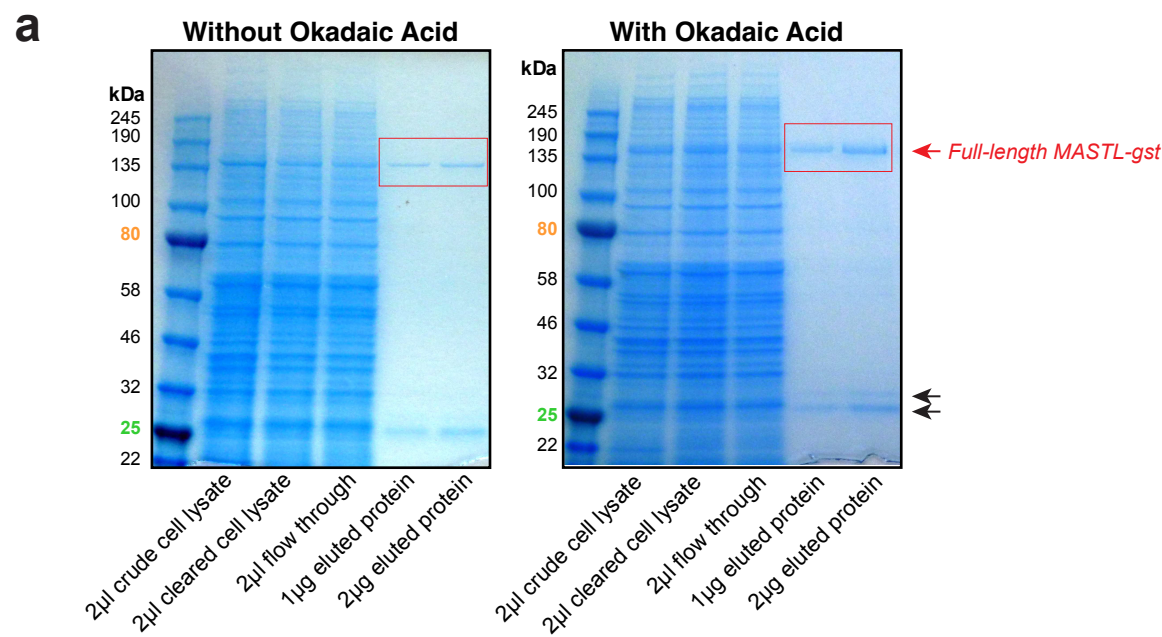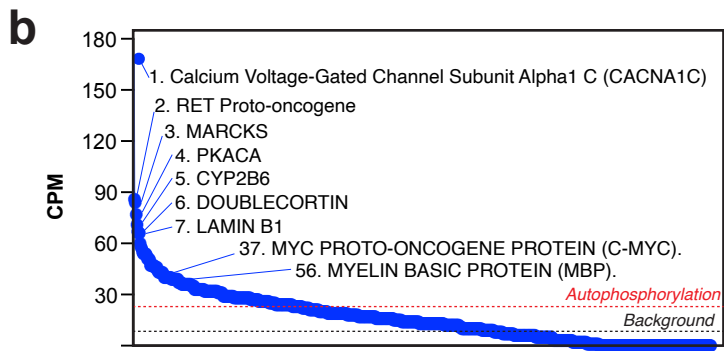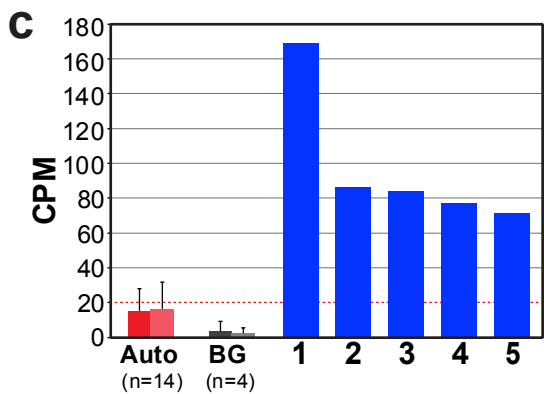

Supplement: Supplementary file 1 — Supplementary Figure S1. [file 41598_2022_14933_MOESM1_ESM.pdf]

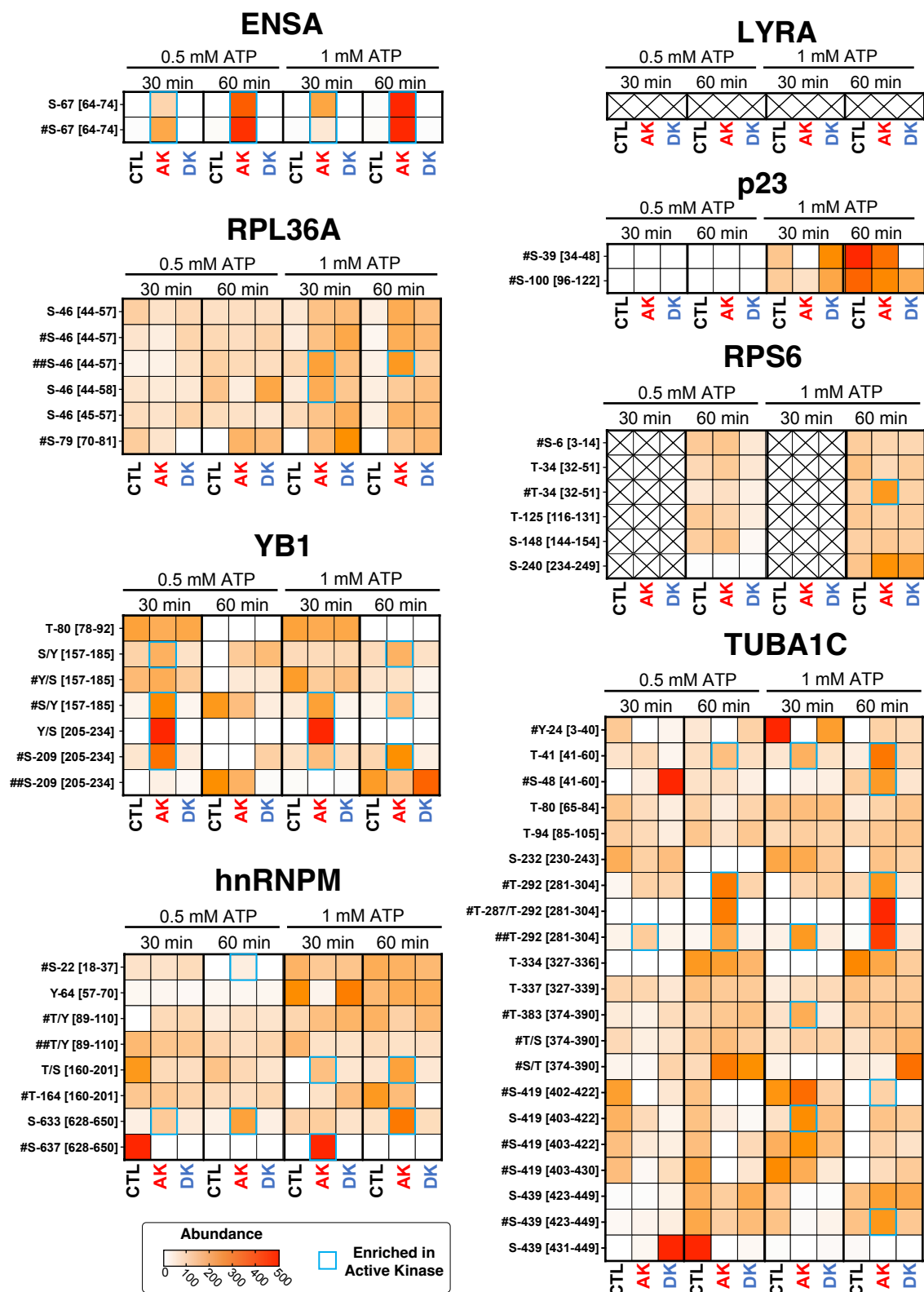

Supplement: Supplementary file 2 — Supplementary Figure S2. [file 41598_2022_14933_MOESM2_ESM.pdf]

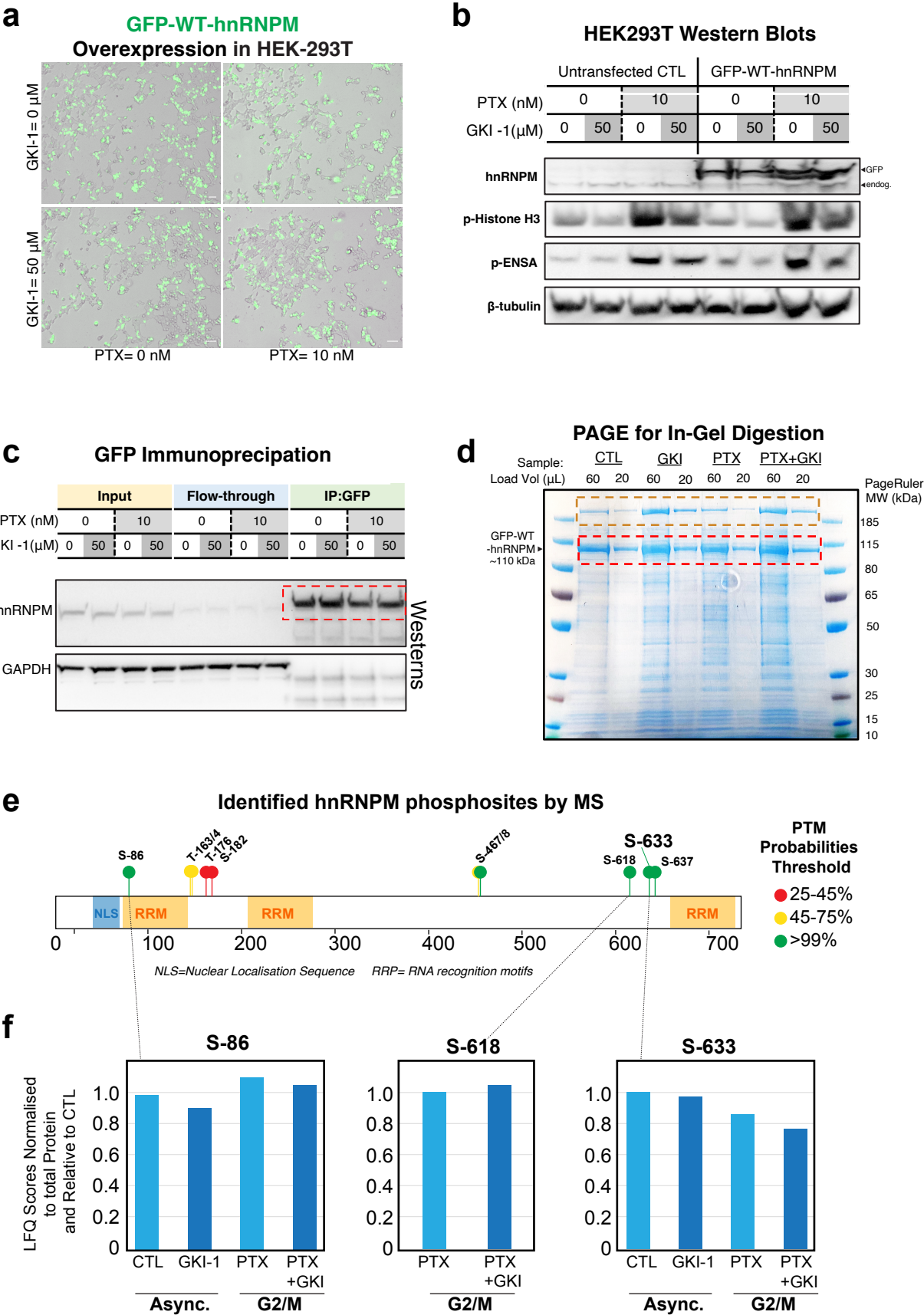

Supplement: Supplementary file 3 — Supplementary Figure S3. [file 41598_2022_14933_MOESM3_ESM.pdf]

Figure 1B

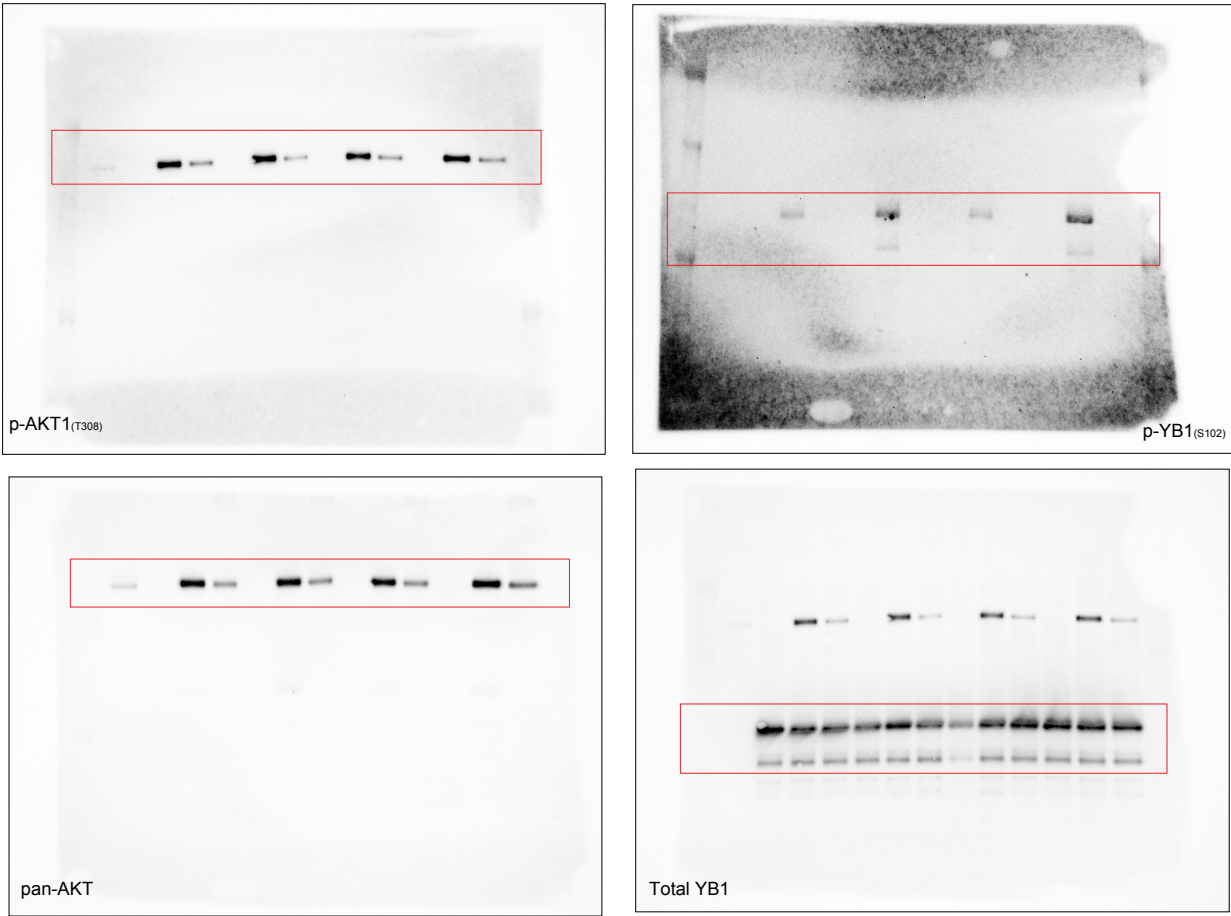

Figure 3D

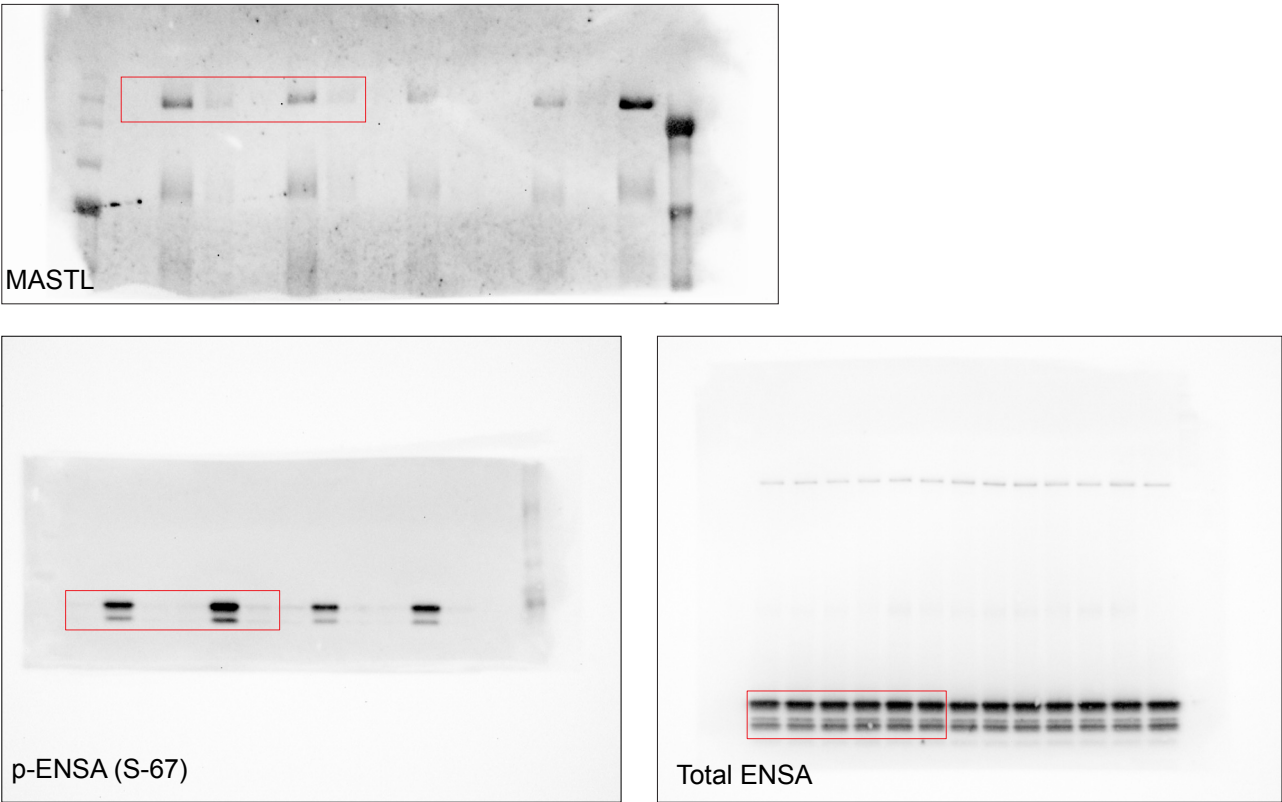

Supplementary Figure S3B

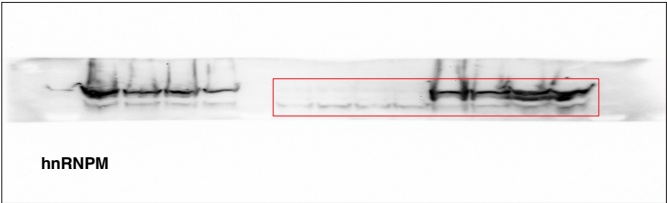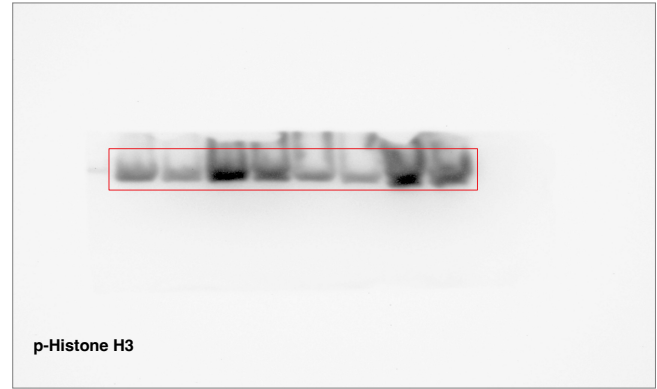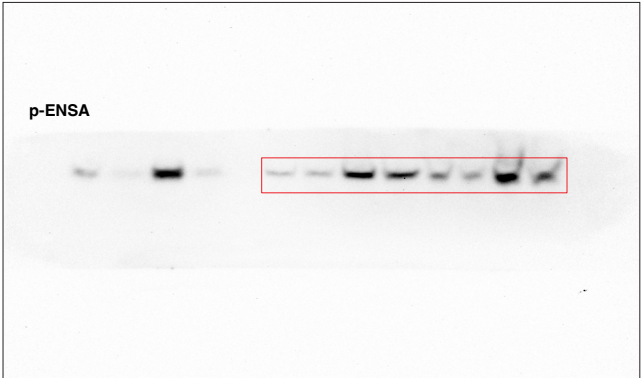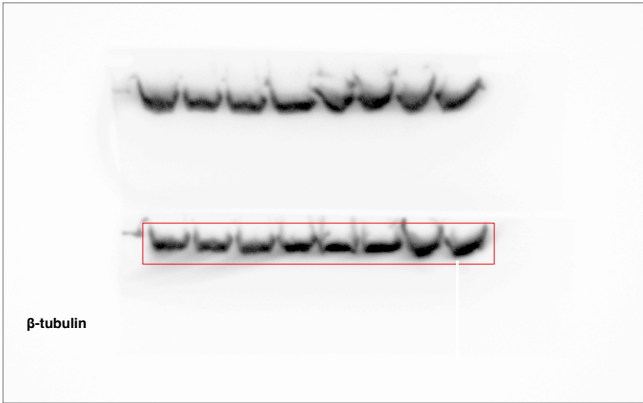

Supplementary Figure S3C

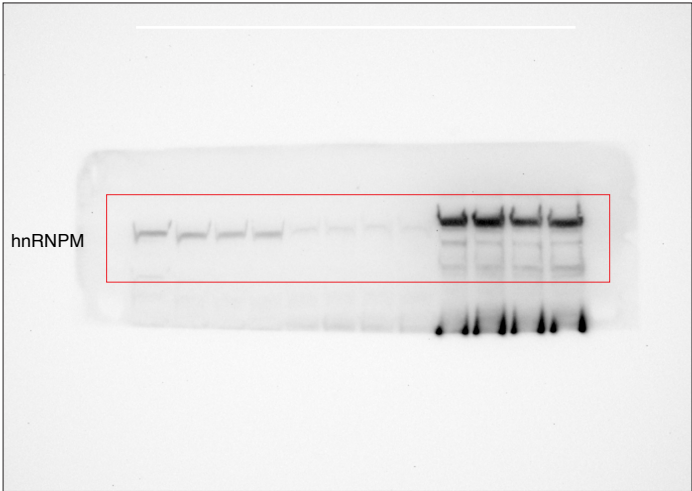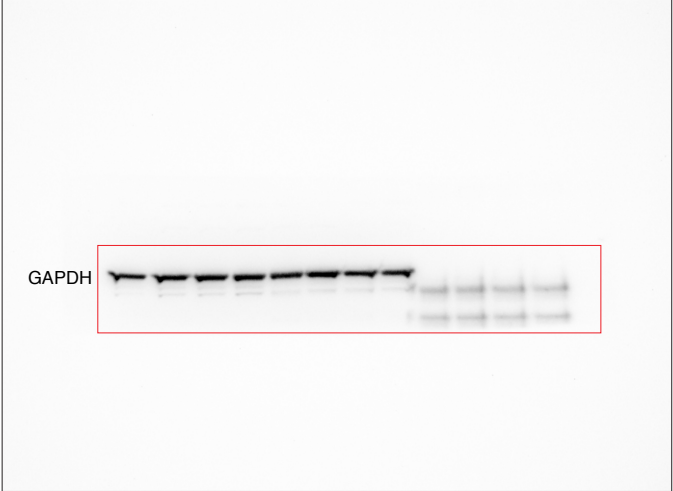

Supplement: Supplementary file 4 — Supplementary Figure S4. [file 41598_2022_14933_MOESM4_ESM.pdf]
