# Supplementary material for: SILAC kinase screen identifies potential MASTL substrates
Source: Sci Rep. 2022 Jun 22;12:10568. doi: 10.1038/s41598-022-14933-0 (PMC9217955; doi:10.1038/s41598-022-14933-0)
Supplement: Supplementary file 13 — Supplementary Legends. [file 41598_2022_14933_MOESM13_ESM.docx]

**SILAC Kinase Screen Identifies Potential MASTL Substrates**

Kamila A Marzec^1#^, Samuel Rogers^2#^, Rachael McCloy^3^, Benjamin L. Parker^4^, David E. James ^5,6^, D. Neil Watkins^7,8^, and Andrew Burgess^1,9*^.

**Supplementary Figure S1: MASTL S/T/Y-Physiological Kinase Substrate Finder Assay**

**(a)** Full-length human MASTL was expressed in Sf9 insect cells with or without 100 nM okadaic acid and purified using GSH-affinity (Glutathione) matrix. Purity was assessed samples by coomassie stained SDS-polyacrylamide gel electrophoresis. **(b)** Radiometric protein kinase assay (^33^PanQinase® Activity Assay) was used for measuring the activity of MASTL on 720 biotinylated peptides known to be phosphorylated *in vivo*. The kinase assays were performed in 384-well polypropylene plates in a 50 μl reaction volume, the subsequent detection of phosphorylated, biotinylated peptides/proteins in 384-well, streptavidin-coated FlashPlate® HTS PLUS (Perkin Elmer, Boston, MA, USA). **(c)** The top 5 phosphorylated peptides from B are shown along with controls for Autophosphorylation (Auto; sample kinase, without substrate) background (BG; EDTA-inactivated MASTL, without substrate) across the two plates are shown separately.

**Supplementary Figure S2: Single-shot *in vitro* kinase screen of potential MASTL substrates.** The abundance of each phospho-site activated in reactions between either no kinase (control, CTL), recombinant active MASTL kinase (active kinase, AK) or heat-treated MASTL kinase (denatured kinase, DK) with recombinant proteins, ENSA, hnRNPM, p23, RPL36A, RPS6, TUBA1C, and YB1. Four reaction conditions were tested (30 min and 60 min incubations, each in the presence of 0.5 mM ATP or 1 mM ATP) for all protein substrates, except LYAR and RPS6 (0.5 mM and 1 mM ATP, 60 min only – absence of 30 min reactions for RPS6 indicated by crossed-out boxes) due to limited protein availability. No phospho-sites were detected on LYAR under any of these conditions. Phospho-site abundances were calculated using label-free quantitation. Square brackets indicate peptide locations within the protein on which each phospho-site was found. Single hashtags denote a co-modification within that peptide, e.g., carbamidomethylation, deamidation, and/or oxidation. Double and triple hashtags denote different combinations of co-modifications within the same peptide (detailed in Supplementary Table S6).

**Supplementary Figure S3: Overexpression of GFP-wt-hnRNPM combined with MASTL inhibition with GKI-1 in HEK-293T cells. (a)** Single frame from live HEK-293T cells expressing GFP tagged full length hnRNPM (GFP-wt-hnRNPM). Cells were transfected using Lipofectamine 3000 for 24 h and images aquired using brightfield and GFP filter using a Plan Flurite 10X objective (NA 0.25) on EVOS FL Auto 2 microscope fited with a GFP filter cube (AMEP4651). Scale bar 50 µm. **(b)** HEK-293T cells were transfected with GFP-wt-hnRNPM for 24 h and compared to untransfected controls (CTL). Cells were co-treated for 8 h with or without 10 nM paclitaxel and with or without 50 µM GKI-1. Cells were then harvested, lysed and analysed by western blot. **(c)** HEK-293T cells over-expressing GFP-wt-hnRNPM were treated as per B. Cells were lysed and immunoprecipitations using anti-GFP primary antibodies were performed. **(d)** Immunoprecipitated GFP-hnRNPM samples from c were subjected to poly acrylamide gel electrophoresis (PAGE). Bands at ~200 kDa (unknown, orange box) and 110 kDa (GFP-wt-hnRNPM, red box) from each lane were excised and combined for each treatment condition, before destaining, reduction-alkylation and in-gel digestion for mass spectrometry. **(f)** Summary of phosphosites on hnRNPM identified by mass spectrometry. **(g)** LFQ scores for phosphosites (S-86 and S-633) were normalised to total protein LFQ scores, and then expressed relative to control (CTL). LFQ scores for S-618 were normalised to total protein and expressed relative to paclitaxel as CTL sample LFQ scores were absent for this site.

**Supplementary Table S1: AKT *in vitro* kinase assay**

Label-free quantitation, Proteome Discoverer v2.3. Each master protein present in the reaction (AKT1 kinase and one substrate protein; blue highlight) is sub-divided into identified phospho-peptides (orange highlight) – Sub-tables can be expanded with (+) icon in left-hand margin. Column information includes accession numbers, %coverage, number of peptide spectral matches (#PSMs), Sequest HT score, KEGG pathways ^50^, abundance ratios and grouped abundances, missed cleavages, confidence scores, co-modifications, and peptide location within the master protein.

**Supplementary Table S2: AKT *in cellulo* kinase assay**

Summary excel file of phosphorylated peptides identified from the AKT2 *in cellulo* kinase screen (n=2). Data was analysed using MaxQuant (v.1.5.3.30) with integrated Andromeda search engine and Perseus.

**Supplementary Table S3: MASTL S/T/Y-Physiological Kinase Substrate Finder Assay**

Excel summary file containing the measured raw data (cpm) for the two 384-well plates labelled "A" and "B" used to measure the activity of purified MASTL kinase against a library of 720 biotinylated peptides using a radiometric protein kinase activity assay (33PanQinase® Activity Assay) using streptavidin-coated FlashPlate® HTS PLUS plates.

**Supplementary Table S4: MASTL *in cellulo* kinase assay**

Summary excel file of phosphorylated peptides identified from the MASTL *in cellulo* kinase screen (n=2). Data was analysed using MaxQuant (v.1.5.3.30) with integrated Andromeda search engine and Perseus.

**Supplementary Table S5: MASTL *in vitro* kinase assay screen**

Label-free quantitation, Proteome Discoverer v2.3. Each substrate analysed separately and displayed in individual tabs. Each master protein present in the reaction (MASTL kinase and one substrate protein; blue highlight) is sub-divided into identified phospho-peptides (orange highlight) – Sub-tables can be expanded with (+) icon in left-hand margin. Column information includes accession numbers, %coverage, number of peptide spectral matches (#PSMs), Sequest HT score, KEGG pathways ^50^, abundance ratios and grouped abundances, missed cleavages, confidence scores, co-modifications, and peptide location within the master protein.

**Supplementary Table S6: *In vitro* kinase assay validation of ENSA and hnRNPM**

Label-free quantitation, Proteome Discoverer v2.3. Each substrate analysed separately and displayed in individual tabs. Each master protein present in the reaction (MASTL kinase and ENSA or hnRNPM; blue highlight) is sub-divided into identified phospho-peptides (orange highlight) – Sub-tables can be expanded with (+) icon in left-hand margin. Column information includes accession numbers, %coverage, number of peptide spectral matches (#PSMs), Sequest HT score, KEGG pathways ^50^, abundance ratios and grouped abundances, missed cleavages, confidence scores, co-modifications, and peptide location within the master protein.

**Supplementary Table S7: Mass spectrometry analysis of purified GFP-wt-hnRNPM treated with and without GKI-1 in HEK-293T cells.**

Label-free quantitation, Proteome Discoverer v2.3. A total of 645 master proteins were identified in the samples, including hnRNPM (77.5 kDa) and myosins (~227 kDa) as the top hits (blue highlight). Sub-tables, which represent the identified peptides of each master protein and their modifications, can be expanded with (+) icon in left-hand margin and are highlighted in orange. Phospho-peptides on hnRNPM are highlighted in bold. Column information includes accession numbers, %coverage, number of peptide spectral matches (#PSMs), Sequest HT score, KEGG pathways ^50^, abundance ratios and grouped abundances, missed cleavages, confidence scores, co-modifications, and peptide location within the master protein.

**Supplementary Table S8: List of Reagents**

A list of all reagents used in this study along with their catalogue number and supplier.
